# Habitual exercise influences carotid artery strain and strain rate, but not cognitive function in healthy middle-aged females

**DOI:** 10.1007/s00421-022-05123-x

**Published:** 2023-01-13

**Authors:** Amy K. Campbell, Alexander J. Beaumont, Lawrence Hayes, Peter Herbert, David Gardner, Louise Ritchie, Nicholas Sculthorpe

**Affiliations:** 1 School of Science, Technology and Health, York St. John University, New York, UK; 2 Sport and Physical Activity Research Institute, University of the West of Scotland, Blantyre, UK; 3 School of Sport, Health and Outdoor Education, Trinity St. David, University of Wales, Carmarthen, UK

**Keywords:** Aging, Exercise, Carotid function, Speckle tracking, Flow-mediated dilation, Cognitive function

## Abstract

**Purpose:**

Aging females are at risk of declining vascular and cognitive function. Exercise can augment both factors independently; however, the influence of exercise on their interdependence is less clearly understood. Ultrasound speckle tracking is a sensitive novel measure of arterial aging but has not previously been used in middle-aged females. We aimed to elucidate the potential interactions between vascular and cognitive variables in active aging females.

**Methods:**

Twelve active (56 ± 5 years; ${\dot{\mathrm{V}}\mathrm{O}}_{2\mathrm{peak}}$: 34.5 ± 6.1 ml.kg.min^−1^) and 13 inactive (57 ± 4 years; 22.8 ± 2.6 ml.kg.min^−1^) healthy middle-aged females were included. Ultrasound speckle tracking assessed short-axis common carotid artery (CCA) compliance via peak circumferential strain (PCS) and strain rate (PSR) at rest, during, and after 3-min isometric handgrip exercise. Flow-mediated dilation (FMD) of the brachial artery was assessed using ultrasound. Cognitive function was measured using Verbal Fluency, Trail Making, Stroop, and Digit Span tests.

**Results:**

PCS (*P* = 0.003) and PSR (*P* = 0.004), were higher in the active cohort. FMD was similar between groups (*P* > 0.05). Minimal differences in cognitive function existed between groups, although the inactive group performed better in one test of animal Verbal Fluency (*P* < 0.01). No associations were observed between PCS, PSR, or FMD with cognitive function (all *P* > 0.05).

**Conclusion:**

This is the first study to assess PCS and PSR in middle-aged females and demonstrates that active middle-aged females exhibit a superior carotid artery profile compared to their inactive counterparts. However, PCS and PSR of the carotid artery may not be linked with cognitive function in middle-aged females.

## Introduction

Aging females are vulnerable to steep declines in vascular health as a result of falling estrogen concentrations, and the resulting reduction in cardio-protection (Mendelsohn 2002). Elevated levels of oxidative stress (Donato et al. 2007) and inflammation (Ungvari et al. 2004) within the peripheral arterial system reduces the bioavailability of vasoactive substances such as nitric oxide (NO) (Taddei et al. 2001), increasing vascular tone and intraluminal pressure (Haynes et al. 1993). Furthermore, age-associated increases in arterial adventitial collagen and calcification, alongside reductions in medial elastin contribute to conduit arterial dysfunction from increasing arterial stiffness (Fleenor et al. 2010). The resulting decrease in Windkessel function augments arterial pulse pressure which can damage local vasculature and distal micro-vessels (Liao et al. 2004), promoting micro-hemorrhages and tissue ischemia (Mitchell et al. 2011). The consequential stiffening of central arteries such as the carotid is believed to impair cerebrovascular function and may contribute to cognitive dysfunction as a result (Wong et al. 2002; Hajjar et al. 2016; Thorin-Trescases et al. 2018). Furthermore, worse peripheral vascular function, as assessed by flow-mediated dilation (FMD), is also reported to associate with poorer cognitive function when assessed in young and older adults (Naiberg et al. 2016; Csipo et al. 2019). Consequently, impaired arterial health could exacerbate normal cognitive adaptations observed with aging (Park et al. 2002).

However, regular physical activity (PA) and exercise are recognized as effective strategies to help ameliorate a range of age-related declines in vascular and cognitive function. For example, middle-aged and older exercisers display superior peripheral endothelial function, as assessed by brachial FMD, than their sedentary and less aerobically fit counterparts (Montero et al. 2014; Campbell et al. 2019). Additionally, healthy older adults who are active, or who have higher aerobic fitness often exhibit higher common and internal carotid artery blood velocity and flow profiles (Azhim et al. 2007; Braz et al. 2017) and better cognitive function (Clarkson-Smith and Hartley 1989; Hillman et al. 2002; Aichberger et al. 2010) when compared to those with lower levels of PA or aerobic fitness. Although it is widely understood that engagement in PA and exercise can result in a variety of positive physiological and neuropsychological health outcomes, little research has investigated the underlying mechanisms responsible for such improvements (Barnes and Corkery 2018).

Existing work have reported positive cognitive function associations with both, cerebrovascular conductance and ${\dot{\mathrm{V}}\mathrm{O}}_{2\mathrm{max}}$ in middle-aged and older females (Brown et al. 2010). More recently, superior cerebrovascular reactivity (CVR), brachial FMD, and cognitive function was observed in endurance trained vs sedentary adults, with CVR and FMD positively associating with cognitive function (Tarumi et al. 2015). Thus, whilst transcranial Doppler is frequently used to measure cerebrovascular function, superior brachial FMD may indirectly represent the cerebrovascular endothelium and potentially, blood–brain barrier integrity (Abbott et al. 2010; Tarumi et al. 2015). These data suggest that an advantageous vascular profile from superior aerobic fitness levels and exercise training may be linked with a delayed onset of age-associated cognitive decline, yet existing literature has been limited to the assessment of cognitive function with cerebrovascular function and brachial artery FMD only. As regular PA may benefit other vascular measures including carotid artery compliance and blood flow (Braz et al. 2017; Talbot et al. 2020), simultaneous assessment of additional vascular measures may be required in an attempt to further explain the potential mechanisms underpinning PA-mediated cognitive benefits. However, no previous study has examined cognitive function with both, central extra-cranial (i.e., carotid artery blood flow, PCS and PSR) and peripheral vascular function (i.e., brachial artery FMD) within the same cohort. Therefore, whilst exercise-induced improvements in FMD, arterial stiffness, and compliance may be linked with cognitive function (Tarumi et al. 2015; Okamoto et al. 2019), currently such associations between both variables can only be implied within the same cohort. Elucidating the potential physiological mechanisms underlying exercise-induced neuropsychological functions may help to better advise optimal exercise-based strategies to reduce age-associated cognitive decline (Barnes and Corkery 2018).

Whilst conventional measures, such as pulse wave velocity (PWV) and beta-stiffness index (β), are commonly used to assess arterial stiffness (Pannier et al. 2002; Tanaka 2017), the recent application of ultrasound speckle tracking may be a superior method of assessing central arterial compliance (Bjallmark et al. 2010). For example, greater arterial expansion {peak circumferential strain (PCS)] and peak rate of expansion [peak strain rate (PSR)] have previously been seen in younger compared to older adults (Bjallmark et al. 2010; Talbot et al. 2020), and in those with higher aerobic fitness levels (Pugh et al. 2018; Talbot et al. 2020), suggesting contemporary assessment techniques are adequate for detecting differences between fitness levels. Furthermore, most studies have investigated exercise-related arterial measures at rest (Tanaka et al. 2000; Shibata et al. 2018; Talbot et al. 2020). However, assessing exercise-induced vascular responses may be beneficial during periods of acute isometric exercise where transient increases in arterial pulsatile pressure occur (Seals 1993). For example, Black et al. (2016) reported reductions and increases in PCS and PSR during and immediately after an acute bout of isometric exercise, respectively, when compared to resting conditions in young healthy males. Assessing common carotid artery (CCA) PCS and PSR during periods of acute physiological stress may therefore provide information regarding potential exercise-induced vascular adaptations compared to only at rest. However, no previous study has used ultrasound speckle tracking to measure PCR and PSR within active and inactive healthy middle-aged females before, during and immediately after acute physiological stress. As middle-aged females are at a high risk of vascular impairment (Moreau et al. 2012; Hildreth et al. 2014), and have a higher risk of cardiovascular-associated cognitive decline than males (Huo et al. 2022), it is important to elucidate the potential links between conventional and novel physiological measures with cognitive performance, to better understand how to delay aging-associated declines in health and well-being.

Therefore, the aims of the study were to: 1) identify whether regularly exercising healthy middle-aged females have superior BA FMD, CCA and internal carotid artery (ICA) blood flow, CCA PCS, PSR, and cognitive function compared to inactive controls; 2) establish whether differences in PCS and PSR exist between groups pre, during and immediately after a 3-min isometric handgrip (IHG) exercise; and 3) determine whether any associations exist between vascular and cognitive variables. It was first hypothesized that the active females would have greater FMD, PCS, PSR, and carotid blood flow profiles in addition to better cognitive function compared to the inactive group. Second, we hypothesized that acute IHG exercise would significantly reduce CCA PCS and PSR during IHG and significantly increase immediately post exercise. It was also hypothesized that associations would exist between superior vascular and cognitive variables.

## Methods

### Ethics approval and recruitment

Ethical approval was granted by the ethics committee at University of Wales, Trinity St. David. Participants were healthy middle-aged females recruited via social media, newspaper advertisements, posters and word-of-mouth within Carmarthenshire, UK. All were provided with information sheets prior to completing a medical questionnaire, physical activity readiness questionnaire (PAR-Q) and providing informed written consent before participation.

### Study design and participants

A cross-sectional design was adopted to identify whether differences in vascular and cognitive function existed between a group of active and inactive middle-aged females. Twenty active and 32 inactive healthy middle-aged females were initially recruited based on self-reported regular or non-regular PA. Participants were divided into active or inactive groups based on whether they self-reported meeting or not meeting the current World Health Organization PA guidelines for adults of 150 min of moderate or 75 min of vigorous PA, and 2 or more days of strength-based exercise per week (World Health Organisation 2020). As not all ‘inactive’ females identified as being completely sedentary, the inactive group within this study have not been classified as ‘sedentary’.

**Fig. 1:**
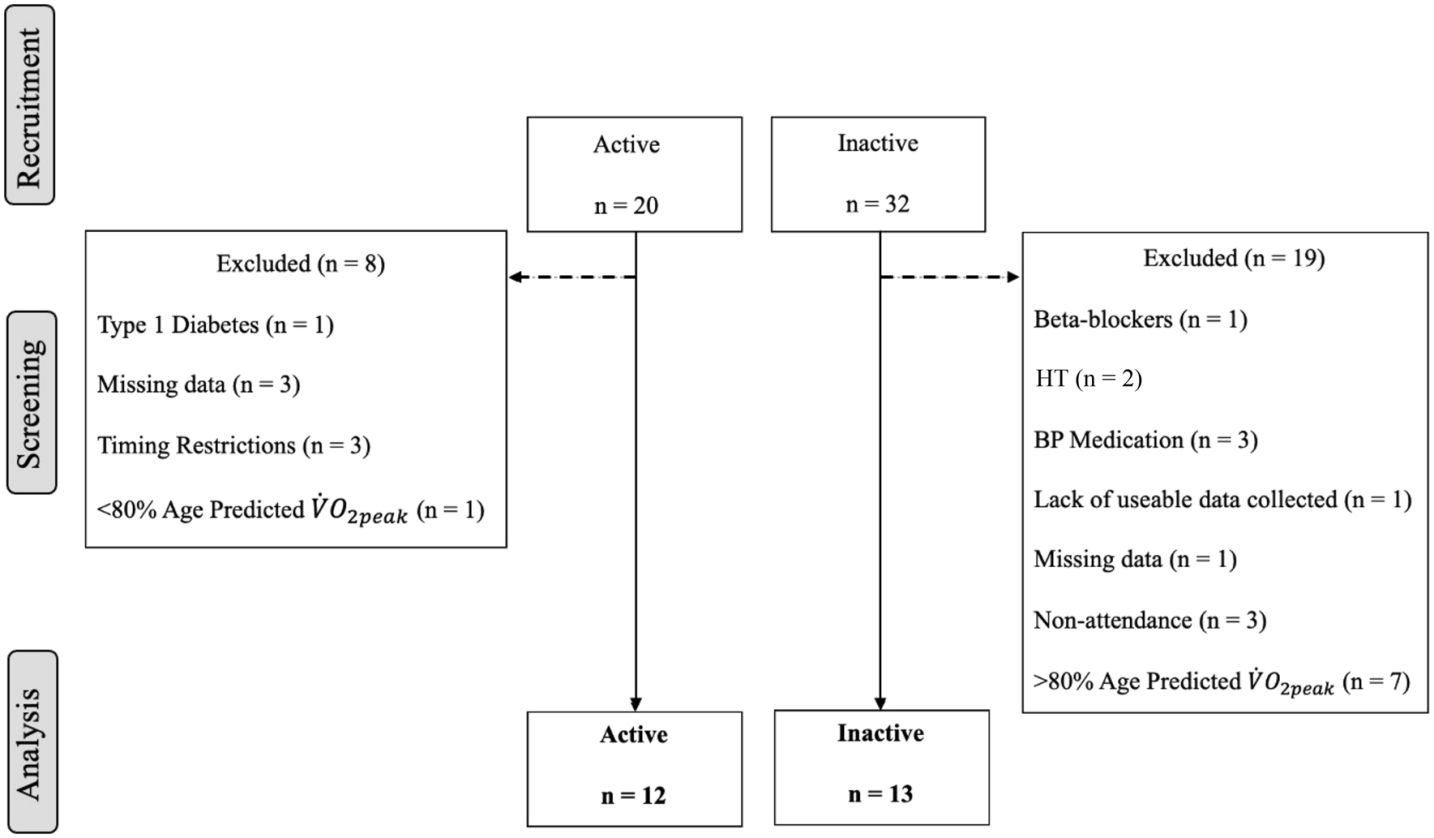
Schematic of participant recruitment, group allocation and reasons for exclusion within the middle-aged cohort. HT hormone therapy, *BP* blood pressure

Medical clearance via written approval from a general practitioner was required prior to participation. Eight and 19 participants were subsequently excluded from the active and inactive groups, respectively for reasons outlined in Fig. 1. Exclusion criteria included use of cardioactive medication, hormone therapy, and conditions which are known to affect vascular health (i.e., cardiovascular diseases and diabetes). Furthermore, active participants with less than 80% and inactive participants with greater than 80% of their age predicted maximum aerobic capacity were also excluded from the study. Due to cross-over in ${\dot{\mathrm{V}}\mathrm{O}}_{2\mathrm{peak}}$ values between groups, this approach was taken to create two distinct groups within the study. Age predicted ${\dot{\mathrm{V}}\mathrm{O}}_{2\mathrm{peak}}$ levels for each participant were calculated based on a large database of aerobic capacity reference data of healthy individuals aged 20 – 90 years (Loe et al. 2013), and a similar approach to create distinct study groups has been used previously in a cohort of middle-aged and older females (Brown et al. 2010). An 80% threshold was chosen as healthy exercise trained and untrained older adults within a recent study investigating CCA PCS and PSR exhibited ${\dot{\mathrm{V}}\mathrm{O}}_{2\mathrm{peak}}$ levels below and above 80% of their age predicted ${\dot{\mathrm{V}}\mathrm{O}}_{2\mathrm{peak}}$, respectively (Talbot et al. 2020). The final groups within this study consisted of 12 active and 13 inactive participants.

Participants were apparently healthy, non-smokers, and did not suffer from overt medical conditions affecting the cardiovascular system. Medications taken by participants consisted of antihistamines and antidepressants, and medications for treating hypothyroidism, migraines and osteoporosis. None were using cardioactive medication or hormone therapy (HT), and all participants verbally confirmed post-menopausal status.

### Protocol

All participants arrived at the laboratory after a 3 h fast and having not consumed alcohol or caffeine for 24 h. Participants were also asked to refrain from strenuous PA 24 h prior to each visit. Body mass and body fat percentage were measured via bioelectrical impedance (Tanita (MC-980MA Plus), Tanita Corporation, Tokyo, Japan), and a stadiometer was used to assess stature shortly after arrival. Participants lay supine for 10 min before being connected to a 3-lead electrocardiogram (ECG) on a Vivid IQ Ultrasound machine (GE Medical, London) for measurement of heart rate (HR). Continuous beat-to-beat blood pressure (BP) was measured using a Finapres monitoring device, of which the cuff was secured to the participant’s third finger of the left hand and positioned level with the heart (Finapres Medical Systems, The Netherlands; Model 1). The beat-to-beat reconstructed finger to brachial BP waveform was stored via the BeatScope Easy software (SMART Medical, UK; Version 02.10 build 004) on a connected computer. Measurements of HR and BP were collected during the carotid ultrasound assessments as described below.

### Vascular assessment

**Fig. 2:**
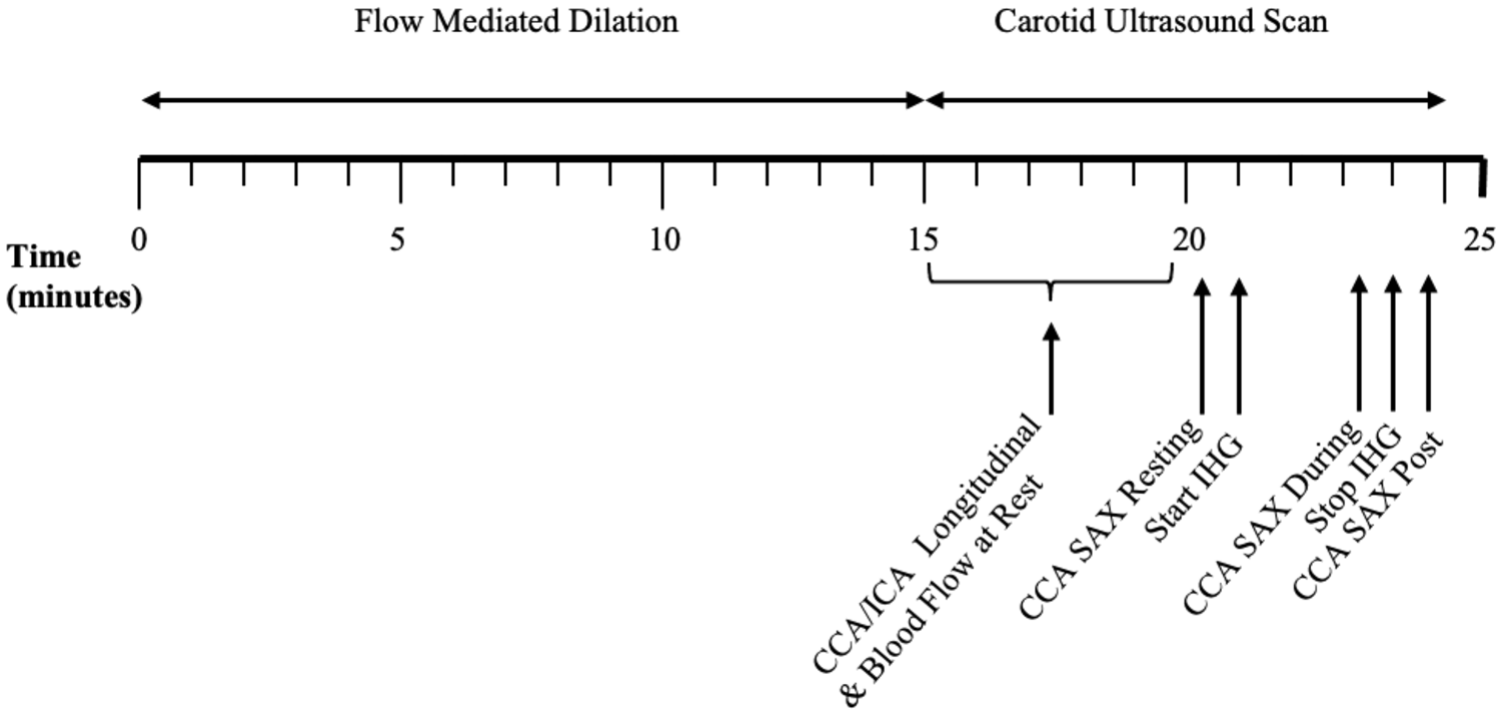
Schematic of the order and timings of the vascular ultrasound protocol. CCA, common carotid artery; ICA, internal carotid artery; SAX, short axis; IHG, isometric handgrip

The order and timings of the vascular ultrasound assessment are presented in Fig. 2. A single researcher collected all vascular ultrasound images (AKC). Image quality of the near and far walls of all vascular measurements was optimized by adjusting the gain, depth, zoom, focus positions, and dynamic range settings. Blood velocity was also measured using Doppler, whereby the Doppler sample gate encompassed the full width of the lumen at an insonation angle of 60 degrees.

### Flow-mediated dilation

The function of the vascular endothelium was assessed via FMD of the BA in accordance with expert consensus guidelines for the protocol (Thijssen et al. 2019). High-resolution B-mode ultrasound imaging was performed using a Vivid IQ (GE Healthcare, London), with a 12 MHz linear array transducer within the active group, and a Terason (uSmart 330, Teratech, Burlington, MA) with a 15L4 Smart Mark transducer within the inactive group. After 10 min of supine rest, a large diameter pneumatic cuff (Hokanson rapid cuff inflator, DE Hokanson Inc., Bellevue, Washington, USA) was secured distally to the antecubital fossa on the right arm, and the whole arm was abducted to approximately 90 degrees. The transducer was positioned longitudinally above the antecubital fossa on the upper arm to locate a straight and parallel segment of the BA.

Duplex longitudinal and Doppler image capture was continuously recorded throughout the FMD protocol. Baseline data were collected for 1 min before rapidly inflating the cuff to supra-systolic pressure (220 mmHg) to occlude blood flow to the lower arm for a 5-min period. BA diameter and blood velocity were subsequently measured for 1 min before, and 5 min after release of the cuff using an automatic edge-detection system (Brachial Analyzer, Medical Imaging Applications LLC, Coralville, IA). BA diameter and blood velocity data were then exported to an excel spreadsheet which was later uploaded to a custom software. The custom software calculated shear stress and arterial diameters averaged over 3-s periods before calculating absolute (mm), relative (%), and shear normalized FMD (%).

### Carotid artery structure and blood flow

All carotid artery imaging was performed using a high-resolution B-mode ultrasound (Vivid IQ) and a 12 MHz linear array transducer (GE Medical, London). Longitudinal images of the common carotid artery (CCA) were measured approximately 1–2 cm inferior to the bifurcation to measure intima-media thickness (IMT). M-mode images of the CCA were recorded over at least three cardiac cycles to identify systolic and diastolic CCA diameter, and Doppler imaging was also used to measure the blood velocity of both the ICA and CCA. Longitudinal images of the ICA were also collected to assess systolic diameter and mean blood velocity.

All CA images were analyzed offline (EchoPAC, GE Healthcare; version 202). The IMT of the posterior CCA wall was measured using the automatic post-processing function over at least a 1 cm straight segment 1–2 cm inferior to the carotid bifurcation. IMT was measured over two consecutive cardiac cycles during diastole and averaged, as recommended by Selzer et al. (2001). Manual callipers measured arterial diameter from the luminal IMT edge of the near and far walls and was averaged over three cardiac cycles. CCA and ICA blood flow per minute was calculated by multiplying the time averaged mean velocity (TAMEAN) with the peak diameter, and then multiplied by 60 as suggested by Blanco (2015). Pulsatility index of the CCA and ICA were calculated at rest using the following equation:

$$Pulsatility\, Index= \frac{Peak\, Systolic\, Velocity-End\, Diastolic\, Velocity}{Time\, Averaged\, Mean\, Flow\, Velocity}.$$

Beat-to-beat BP was recorded simultaneously with each resting carotid image using customized markers within the BeatScope Easy software. HR was also collected alongside each ultrasound image via the 3-lead ECG connected to the Vivid IQ ultrasound. BP and HR were subsequently averaged across resting images to provide resting values.

### Carotid artery peak circumferential strain and strain rate

PCS and PSR of the CCA were assessed at rest, during, and immediately after a 3-min isometric handgrip (IHG) exercise. The assessment was made through a short-axis (SAX) image of the CCA, taken approximately 1 cm inferior to the bifurcation at a depth of 4 cm and frame rate of 106.9 frames per second. A high frame rate was attained through standardized manipulation of the ultrasound’s width, depth, focal point, and frequency settings. A 3-min IHG contraction at 40% of maximal effort was chosen as it has previously been shown as a sufficient intensity to elicit physiological stress (Weiner et al. 2012), whereby higher intensities are not reported to produce greater BP responses (Seals 1993).

After resting measurements, participants gripped a hand dynamometer (Takei T.K.K.5001 Grip A, Takei Scientific Instruments Co. Ltd, Tokyo, Japan) at 40% of their maximum, which was measured after completing one maximal effort grip prior to the assessment. One maximal effort grip was performed prior to calculating 40% to limit the influence of localized fatigue prior to the 3-min isometric contraction. SAX CCA images were recorded at the end of the 3-min contraction, and within 20 s after release of the hand dynamometer, as similarly performed by Black et al. (2016). To ensure the same segment of the CCA was imaged during each timepoint, the ultrasound transducer remined in the same position throughout the 3-min contraction. BP and HR values were also measured simultaneously with each carotid image taken during and immediately after the 3-min IHG exercise.

The short-axis apex (SAX-AP) 2D strain option within the EchoPAC software was selected to measure CCA PCS and PSR. The region of interest (ROI) was aligned immediately posterior to any visible CCA IMT, which tracked ultrasound speckle movement frame-by-frame across the cardiac cycle. The software segmented the CCA cross-section into six sections, whereby the values for each were automatically averaged together to provide global values for PCS, PSR, and recoil rate. Global PCS, PSR, and recoil rate values of the mid-section were recorded only if 4 or more segments of the wall were successfully tracked by the software. All 6 segments of the arterial wall were successfully tracked within > 85% of images.

The analyzed EchoPAC files were imported into a custom software (Vascular Strain Toolkit, V0.5beta) which extracted the required vascular values using a 1000 point cubic spline within both systole and diastole to measure peak expansion during systole (PCS, %), peak rate of expansion during systole (PSR, 1/s), and recoil rate (1/s) of the CCA during diastole. Pulse transit time (PTT) was also measured from the software, calculated as the time delay between left ventricular ejection as indicated from the ECG the QRS complex, and the onset of CCA expansion measured via ultrasound (Sculthorpe et al. 2020) to represent arterial stiffness. PCS, PSR, recoil rate, and PTT were corrected for physiologically relevant timings for carotid mechanics, as reported by Sculthorpe et al. (2020), and were subsequently averaged over two cardiac cycles. The coefficient of variation of strain-derived resting vascular measures within our lab are as follows: PCS, 14.6%; PCR, 13.4%; recoil rate, 15.8%; and PTT, 9%.

Systolic and diastolic SAX arterial diameters from one of the included PCS and PSR cardiac cycles were assessed at each time point using manual callipers from the intima-lumen interface of the anterior and posterior walls.

### Conventional carotid artery stiffness

Conventional measurement of arterial stiffness was also calculated to identify whether CCA stiffness changed from pre, during and immediately after the IHG exercise. Conventional stiffness measures Petersons Elastic Modulus (Ep) and Beta-1 stiffness index (β) were calculated using the following equations (Bjallmark et al. 2010):

1 $$\mathrm{\beta \, stiffness \, index }(\mathrm{cm}2/\mathrm{kPa})= \frac{In \left(SBP/DBP\right)}{(DiamSYS-DiamDIAS)/DiamDIAS},$$

2 $$\mathrm{Petersons's \, Elastic \, Modulus } \, (\mathrm{Ep}) \, (\mathrm{kPa})= \frac{(SBP-DBP)}{(DiamSYS-DiamDIAS)/DiamDIAS}.$$

where In = natural logarithm, SBP = systolic blood pressure, DBP = diastolic blood pressure, DiamSYS = diameter of the artery during systole, DiamDIAS = diameter of the artery during diastole.

### Cognitive function assessment

Prior to commencing the cognitive assessments, participants completed the Centre for Epidemiologic Studies Depression Scale (CES-D) and the Warwick–Edinburgh Mental Well-being Scale (WEMWBS). Cut-off scores ≥ 16 and < 44.5 were used to screen for individuals at risk of depression and low mental well-being, respectively (Bianco 2012), and data were subsequently removed for such participants.

The same researcher administered all cognitive tests to participants, which consisted of the following:

### Trail making A and B

To assess processing time during Trail A, participants were presented with one sheet of paper containing circles numbered 1 – 25 and were asked to draw a line between each in numerical order as quickly as possible (Lim et al. 2013). Trail B consisted of circles numbered 1 – 13 and letters A – L. Participants were required to draw a line between numbers and letters in numerical and alphabetical order (1 – A – 2 – B – 3 – C, etc.) to assess executive function (Lim et al. 2013).

### Digit span forwards and backwards

Participants were asked to repeat lists of numbers in the same order in which they were read out. Difficulty ranged from a 2- to a 12-number sequence, and participants were required to correctly repeat at least 5 of 6 sequences at each difficulty stage before progressing. Backwards span required participants to recite sequences ranging from 2 to 10 numbers in the reverse order from which they were read out. In each test, Digit span was determined by the last completed stage whereby at least 5 out of 6 sequences were correctly repeated. Digit span forward and backwards are commonly used for assessing short-term memory (Jones and Macken 2015) and attention (Lim et al. 2013), respectively.

### Verbal fluency

To assess executive function (Lim et al. 2013), participants named as many animals as possible (Animal fluency) and words beginning with ‘s’ (Letter ‘s’ Fluency) in two separate 1-min rounds.

### Stroop test

The Stroop test consisted of 4 separate assessments printed on paper. Participants read aloud the names of colors printed in black ink for Stroop 1, the names of colors printed in their respective colors for Stroop 2, and the colors of printed symbols for Stroop 3. During Stroop 4, participants were presented with paper containing names of colors printed in ink that did not match the color name (e.g., ‘green’ printed in blue ink). Participants were asked to read aloud the color of the ink the word was printed in. Each test contained 12 printed words/symbols, and the objective was to complete each task as quickly as possible. The Stroop test is commonly used to assess information processing speed (Coetsee and Terblanche 2017) and executive function (Lim et al. 2013).

### Aerobic capacity assessment

Aerobic capacity was determined using open circuit spirometry using a Cortex II Metalyser 3B-R2 (Cortex, Biophysik, Leipzig, Germany). Participants performed a 5-min warm-up on a treadmill set at 5 km/hr and a 2% gradient. Following a 3-min recovery, participants were fitted with a facemask before completing the modified Bruce Protocol to volitional exhaustion (Bruce et al. 1973). The incremental protocol consisted of 3-min stages beginning at 2.74 km/hour with a 10% gradient. Stage-by-stage increments in treadmill gradient (%) and speed (km/h) followed guidelines recommended by Bruce et al. (1973). Oxygen uptake (VO_2_), carbon dioxide production (VCO_2_), respiratory exchange ratio and ventilation were displayed continuously. HR was recorded every 5 s using short-range telemetry (Polar T31, Kempele, Finland). Participants indicated perceived exertion using the Borg RPE scale (Borg 1970), which was recorded during the last 10 s of each 1-min stage. Breath by breath data were sampled and transferred to a PC for real-time display. Coefficient of variation for the determination of ${\dot{\mathrm{V}}\mathrm{O}}_{2\mathrm{peak}}$ in our laboratory is < 3.0%. Subsequently measured ${\dot{\mathrm{V}}\mathrm{O}}_{2\mathrm{peak}}$ was assessed as a proportion of age predicted ${\dot{\mathrm{V}}\mathrm{O}}_{2\mathrm{peak}}$ (Loe et al. 2013).

### Statistical analysis

Data were tested for normality and equal variances using a Shapiro–Wilk and Levene’s test, respectively. An unpaired student’s t-test or Mann–Whitney U test was used to identify resting differences between the active and inactive groups. A two-way repeated-measures ANOVA (group x time) analyzed variables measured between groups at rest, during and immediately after the IHG exercise. When significance was present for time or interaction, Tukey’s post hoc test was used to identify pairwise comparisons. A correlation analysis was also conducted between vascular and cognitive variables which differed between groups at the P < 0.05 level, and between vascular variables and aerobic capacity. Pearson’s and Spearman’s correlation coefficients were used when correlations which met, or failed to meet normality assumptions, respectively. Effect sizes for the main resting vascular and cognitive outcome variables were calculated as Cohen’s D (d) where values of 0.2, 0.5 and 0.8 represented a small, medium and large effect, respectively (Cohen 1988). The effect sizes of the main vascular variables measured pre, during and post IHG exercise were calculated as Eta squared (η^2^) where values of 0.01, 0.06 and 0.14 represented a small, medium and large effect, respectively. All data are displayed as mean ± standard deviation (SD), and an α level of < 0.05 was chosen to identify the presence of statistical significance. All statistical analysis was performed using the Jamovi statistical software, version 0.9 (2019).

## Results

### Missing data

Anthropometric and aerobic capacity data for 1 active participant could not be retrieved, and BP data were also irretrievable for 1 inactive and 3 active participants. Due to poor image quality, CA and ICA images for 1 inactive and 1 active participant were excluded from the analysis, respectively. Due to technical difficulties, 1 active participant did not complete the IHG exercise. FMD results are also absent for 2 active participants due to: 1) poor image quality; and 2) voluntary withdrawal from the FMD assessment.

### Participant characteristics

The characteristics of the active and inactive groups are contained within Table 1. There were no differences in age, height or hemodynamic variables between the active and inactive groups (*P* > 0.05). The active participants had higher ${\dot{\mathrm{V}}\mathrm{O}}_{2\mathrm{peak}}$, age predicted ${\dot{\mathrm{V}}\mathrm{O}}_{2\mathrm{peak}}$ percentage, and lower body mass, body fat percentage and BMI compared to the inactive middle-aged females (Table 1).

**Table 1:** Participant characteristics, hemodynamics, vascular structure and function of the active and inactive groups during resting conditions

Measured variables	Active	Inactive	*P*-value
Age (years)	56 ± 5	57 ± 4	0.730
${\dot{\mathrm{V}}\mathrm{O}}_{2\mathrm{peak}}$(ml.kg.min^−1^)	34.5 ± 6.1	22.8 ± 2.6	< 0.001
Age Predicted ${\dot{\mathrm{V}}\mathrm{O}}_{2\mathrm{peak}}$ (%)	102.6 ± 16.1	67.6 ± 6.4	<0.001
Height (m)	1.62 ± 0.06	1.63 ± 0.06	0.640
Mass (kg)	64.2 ± 8.8	78.5 ± 11.4	0.002
BMI (kg/m^2^)	24.6 ± 4.1	29.6 ± 4.7	0.009
Body fat (%)	26.6 ± 7.6	40.1 ± 4.2	< 0.001
SBP (mmHg)	126 ± 26	140 ± 17	0.160
DBP (mmHg)	66 ± 16	73 ± 12	0.260
MAP (mmHg)	86 ± 19	94 ± 14	0.300
PP (mmHg)	61 ± 14	65 ± 12	0.400
Common carotid artery			
Systolic longitudinal diameter (cm)	0.58 ± 0.04	0.60 ± 0.04	0.280
Diastolic longitudinal diameter (cm)	0.51 ± 0.03	0.53 ± 0.04	0.030
Posterior intima-media thickness (mm)	0.63 ± 0.09	0.62 ± 0.10	0.790
Blood flow (ml.min^−1^)	442 ± 71	509 ± 67	0.019
Pulsatility index	1.9 ± 0.4	1.7 ± 0.2	0.026
Peak recoil rate (1/s)	− 0.15 ± 0.05	− 0.14 ± 0.04	0.470
Pulse transit time (ms)	54.3 ± 17.4	45.0 ± 13.1	0.158
β (mm^2.^kPa^−1^)	6.6 ± 3.0	7.8 ± 3.4	0.758
E_p_ (kPa)	78 ± 38	105 ± 43	0.169
Internal carotid artery			
Systolic longitudinal diameter (cm)	0.42 ± 0.05	0.44 ± 0.06	0.260
Blood flow (ml min^−1^)	353 ± 125	416 ± 110	0.420
Pulsatility index	1.4 ± 0.4	1.2 ± 0.1	0.060
Brachial artery			
Baseline diameter (cm)	0.36 ± 0.05	0.35 ± 0.04	0.540
FMD (%)	6.6 ± 2.7	7.0 ± 3.3	0.760
Shear normalized FMD × 10^–5^ (%)	2.6 ± 13.7	7.6 ± 12.5	0.220
FMD time to peak (s)	77.6 ± 22.3	120.5 ± 71.8	0.080

Data are presented as mean ± standard deviation, and significant *P*-values are displayed in bold. ${\dot{\mathrm{V}}\mathrm{O}}_{2\mathrm{peak}}$, maximum aerobic capacity; *BMI* body mass index; *HR* heart rate; *SBP* systolic blood pressure; *DBP* diastolic blood pressure; *MAP* mean arterial pressure; *PP* pulse pressure; *E*_*p*_, Peterson’s Elastic Modulus; *FMD* flow-mediated dilation

### Vascular measures at rest

The inactive females displayed lower pulsatility index of the CCA (*P* = 0.026; mean difference (MD) = − 0.292; 95% CI = −0.5 45, −0.039) and higher CCA blood flow (*P* = 0.019; MD = 77 ml.min^−1^; 95% CI = 18.1, 122). No other differences in carotid or brachial artery structure and function were observed between groups when analyzed at the *P* < 0.05 level (Table 1).

### Vascular measures during and after isometric handgrip exercise

All hemodynamic variables increased during and immediately after the three-minute IHG exercise compared to resting conditions (*P* < 0.001; Table 2), with a group effect of HR demonstrating lower HR in the active group (*P* = 0.002; η^2^ = 0.33; MD = 11.9 b**.**min^−1^; 95% CI = − 5.9, –7.77). No interaction effects were observed for hemodynamic values.

**Table 2:** Hemodynamic and common carotid artery variables

Variable	Active			Inactive			Main Effect *P*-Value		
	Rest	Handgrip	Post-Handgrip	Rest	Handgrip	Post-Handgrip	Time	Group	Interaction
Hemodynamics									
HR (b.min^−1^)	55 ± 6	57 ± 8	57 ± 7	64 ± 8	70 ± 10	70 ± 10	<0.001	0.002	0.102
SBP (mmHg)	126 ± 26	150 ± 19	147 ± 15	140 ± 17	160 ± 26	154 ± 24	<0.001	0.267	0.576
DBP (mmHg)	66 ± 16	78 ± 11	74 ± 9	73 ± 12	82 ± 16	79 ± 15	<0.001	0.342	0.676
MAP (mmHg)	86 ± 19	102 ± 12	98 ± 9	94 ± 14	108 ± 18	104 ± 17	<0.001	0.333	0.872
PP (mmHg)	61 ± 14	72 ± 14	73 ± 14	65 ± 12	77 ± 15	75 ± 13	<0.001	0.475	0.683
Common carotid artery									
Peak recoil rate (1/s)	–0.15 ± 0.05	–0.16 ± 0.06	–0.17 ± 0.04	–0.14 ± 0.04	–0.14 ± 0.05	–0.14 ± 0.04	0.556	0.141	0.587
Pulse Transit Time (ms)	54.3 ± 17.4	49.4 ± 17.5	46.9 ± 14.9	45.0 ± 13.1	48.1 ± 18.4	43.8 ± 12.7	0.473	0.370	0.530
Systolic SAX Diameter (cm)	0.60 ± 0.09	0.60 ± 0.08	0.60 ± 0.08	0.60 ± 0.05	0.62 ± 0.05	0.61 ± 0.06	0.581	0.789	0.053
Diastolic SAX Diameter (cm)	0.55 ± 0.08	0.54 ± 0.07	0.55 ± 0.07	0.55 ± 0.05	0.56 ± 0.04*	0.57 ± 0.05*	0.026	0.716	0.008
Conventional stiffness indices									
β stiffness index (mm^2^.kPa^−1^)	6.6 ± 3.1	7.3 ± 3.0	8.4 ± 3.5	7.8 ± 3.4	9.7 ± 3.0	11.0 ± 6.8	0.072	0.111	0.444
E_p_ (kPa)	78 ± 38	107 ± 48	119 ± 54	105 ± 43	149 ± 57	167 ± 114	0.013	0.069	0.486

Data are presented as means (± standard deviations) at rest, during and immediately after the 3-min isometric handgrip exercise. *P*-values for the main effects of time point (rest, during the 3-min IHG and post IHG), visit (pre to post intervention), and a time * visit interaction are reported, and significant values are displayed in bold. An asterisk (*) represents a significant post hoc difference from inactive resting conditions, as calculated by a time * group interaction. *HR* heart rate; *SBP* systolic blood pressure; *DBP* diastolic blood pressure; *MAP*, mean arterial pressure; PP pulse pressure; *SAX* short axis; *E*_*p*_, Peterson’s Elastic Modulus; *FMD* flow-mediated dilation

**Fig. 3:**
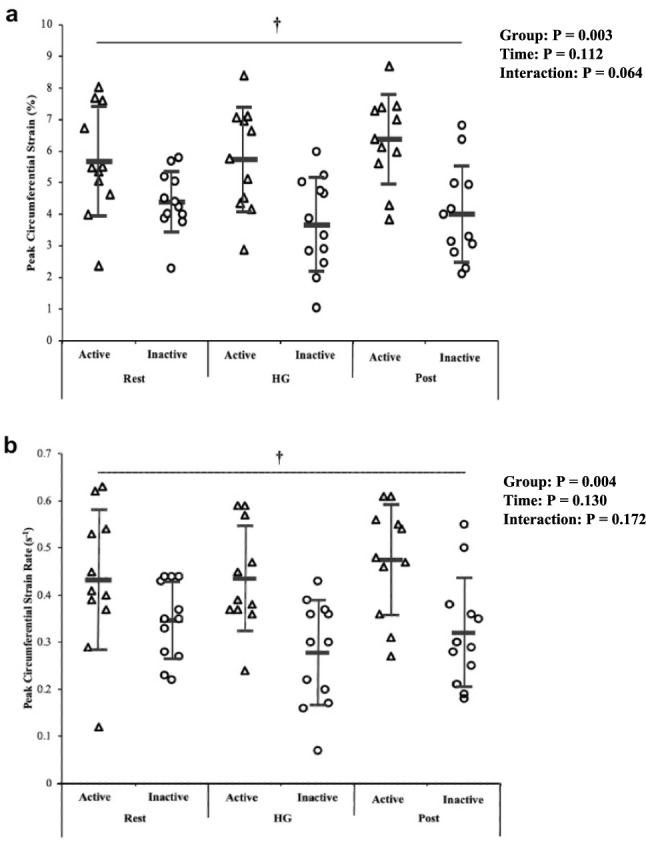
Active and inactive group differences in peak circumferential strain (%; **a**), peak strain rate (1/s; **b**) at rest, during and immediately after the 3-min handgrip (IHG) exercise. Data are presented as individual data points, means ± standard deviations. †  A significant main group effect in favor of the active cohort at *P* < 0.01

Despite no time or interaction effect, there was a significant between group effect for PCS (*P* = 0.003; η^2^ = 0.30; MD = 1.91%; 95% CI = 1.20, 2.61) and PSR (*P* = 0.004; η^2^ = 0.26; MD = 0.13 1/s; 95% CI = 0.08, 0.19), whereby the active cohort exhibited superior values (Fig. 3). There were no other significant group or time effects for recoil rate during diastole or conventional stiffness variables E_p_ and β at the *P* < 0.05 level (Table 2), although a significant effect for time was observed in E_p,_ increasing post IHG compared to pre-IHG measures (*P* = 0.013; η^2^ = 0.072; Table 2).

A significant effect for time was also observed for diastolic SAX diameter, whereby diameter decreased during the IHG compared to rest (*P* = 0.026; η^2^ = 0.004; Table 2). Additionally, a significant group*time interaction effect (η^2^ = 0.006) was also observed for diastolic SAX diameter, showing reductions in the untrained group during (*P* = 0.002) and after the IHG exercise (*P* = 0.02; Table 2) compared to rest.

### Associations between variables

**Fig. 4:**
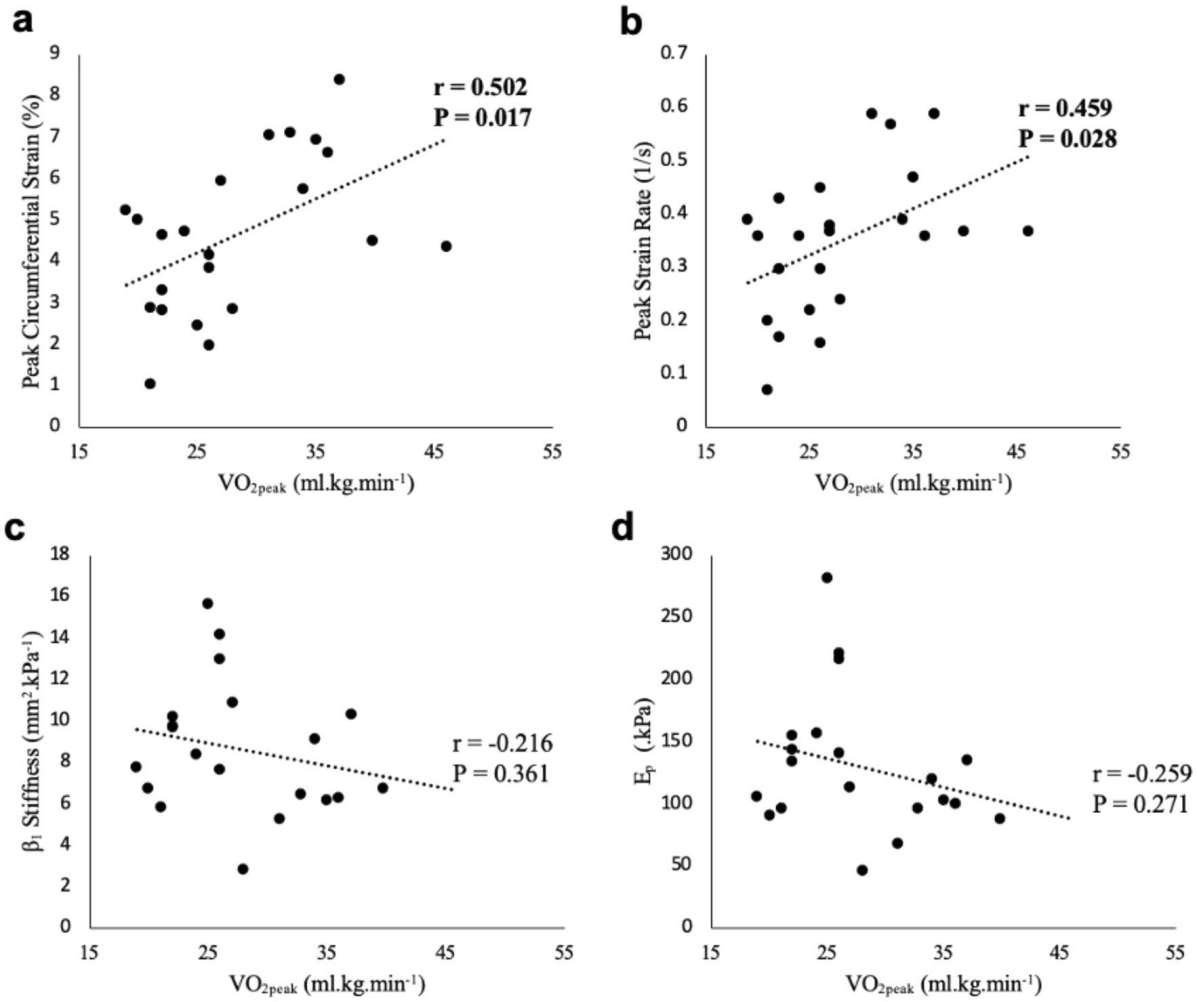
Correlation analysis between measured ${\dot{\mathrm{V}}\mathrm{O}}_{2\mathrm{peak}}$ values and PCS (**a**), PSR (**b**), β (**c**) and E_p_ (**d**) when measured during the 3-min IHG exercise. Significant correlation coefficients and their respective *p* values are displayed in bold

Associations were present between ${\dot{\mathrm{V}}\mathrm{O}}_{2\mathrm{peak}}$ with PCS during IHG (*r* = 0.502, *P* = 0.017), PCS post IHG (*r* = 0.515, *P* = 0.012), and PSR during IHG (*r* = 0.459, *P* = 0.028; Fig. 4). Similarly, associations between age predicted ${\dot{\mathrm{V}}\mathrm{O}}_{2\mathrm{peak}}$ and PCS during IHG (*r* = 0.515, *P* = 0.014), PCS post HG (*r* = 0.536, *P* = 0.008), PSR during IHG (*r* = 0.476, *P* = 0.022) and PSR post IHG (*r* = 0.430, *P* = 0.04) also existed. However, no associations between conventional stiffness measures and ${\dot{\mathrm{V}}\mathrm{O}}_{2\mathrm{peak}}$ were observed (*P* > 0.05; Fig. 4).

CCA PI was also significantly associated with resting PCS (*r* = 0.669, *P* < 0.001) and PSR (*r* = 0.765, *P* < 0.001), as was ICA PI with resting PCS (*r* = 0.695, *P* < 0.001) and PSR (*r* = 0.737, *P* < 0.001). CCA flow was associated with resting PCS (*r* = − 0.475; *P* = 0.031) and PSR (*r* = − 0.439, *P* = 0.041). No significant correlation was observed between ICA flow and strain measures PCS and PSR. Inverse correlations were present between PCS with E_P_ (*r* = − 0.283, *P* = 0.026) when collated across all three timepoints.

### Cognitive function assessment

Three inactive individuals were removed from the cognitive analysis as they identified at risk of depression and low well-being during the CES-D and WEMWBS tests, respectively. Three active individuals were also removed as two identified at risk of depression, and one for low well-being. The decision was made to exclude said participants from the cognitive analysis as the presence of depression has previously been shown to have a detrimental impact on the cognitive function (Lim et al. 2013; Rock et al. 2014). The only cognitive variable to differ between groups at the *P* < 0.05 level was animal fluency, which was higher in the inactive cohort (*P* = 0.002; Cohen’s *d* = 1.65; MD = 5.7 words; 95% CI = 2.5, 9.0; Table 3). An inverse association existed between animal fluency performance with both, measured (*r* = − 0.533; *P* = 0.016) and age predicted ${\dot{\mathrm{V}}\mathrm{O}}_{2\mathrm{peak}}$ (*r* = − 0.576; *P* = 0.008). However, no significant correlations existed between animal fluency performance and vascular measures.

**Table 3:** Cognitive test battery results

Cognitive Test	Active (*n* = 11)	Inactive (*n* = 18)	*P*-value
Trail making			
Trail A (s)	26.5 ± 6.9	26 ± 7.9	0.9
Trail B (s)	55.9 ± 12.8	63.6 ± 21.4	0.36
Stroop Test			
Stroop 1 (s)	4.7 ± 0.8	4.8 ± 1.2	0.82
Stroop 2 (s)	4.9 ± 0.9	4.6 ± 0.8	0.45
Stroop 3 (s)	5.6 ± 0.9	6.4 ± 1.1	0.1
Stroop 4 (s)	11.8 ± 3.2	9.0 ± 3.5	0.08
Verbal fluency			
Animals (*n*)	12 ± 3	18 ± 4	**0.002**
Letter ‘s’ fluency (*n*)	11 ± 4	11 ± 4	0.7
Digit span			
Forwards (*n*)	7 ± 2	7 ± 1	0.84
Backwards (*n*)	5 ± 1	4 ± 2	0.49

Data are presented as means (±SD), and significant *P*-value are displayed in bold

## Discussion

This study aimed to investigate whether differences existed in CCA PCS and PSR, FMD and cognitive function between physically active versus inactive healthy middle-aged females, and whether a relationship between potential vascular and cognitive benefits exists. We also aimed to identify whether CCA PCS and PSR differed between groups when measured before, during and after a 3-min IHG exercise within this healthy middle-aged cohort. The main findings are that active middle-aged females exhibited greater levels of PCS and PSR before, during and immediately after the IHG exercise compared to their inactive counterparts. These findings support previous literature which report that CCA PCS and PSR are a more discriminant measure of central vascular dynamics than conventional stiffness measures (Bjallmark et al. 2010; Pugh et al. 2018). However, few other differences in vascular structure were present between groups, and one test of executive function (animal fluency) was superior in the inactive cohort. Furthermore, no association between animal fluency and vascular measures was observed. Consequently, regular participation in PA and exercise appears to have a positive impact on selective components of vascular function within our cohort of healthy middle-aged females, yet with minimal differences in cognitive function.

### Carotid artery

The current study is the first to investigate the ability of ultrasound speckle tracking to identify differences in CCA PCS and PSR of active and inactive middle-aged females. The active females demonstrated superior PCS and PSR compared to their inactive counterparts. Given that aging has shown to reduce CCA PCS and SR (Bjallmark et al. 2010; Yuda et al. 2011; Park et al. 2012; Rosenberg et al. 2018; Talbot et al. 2020), the findings of the present study suggest that participation in regular exercise may ameliorate declines in intrinsic vascular health in middle-aged females. These findings concur with recent work identifying greater PCS and PSR in middle-aged male runners when compared to their non-running counterparts (Talbot et al. 2020). Furthermore, the significant associations between measured and age predicted ${\dot{\mathrm{V}}\mathrm{O}}_{2\mathrm{peak}}$ with measures of PCS and peak PSR further support that central arterial elasticity and compliance may be influenced by cardiorespiratory fitness levels, as reported previously (Pugh et al. 2018). Although, a genetic component exists with the trainability of ${\dot{\mathrm{V}}\mathrm{O}}_{2\mathrm{peak}}$ (Bouchard et al. 1999), and while the active females in this study did demonstrate superior aerobic fitness than the inactive, our study extends previous work by differentiating groups primarily on reported exercise status. Consequently, our efforts to minimize the sole contribution of aerobic fitness has further strengthened the proposition that regular exercise is an important factor for greater CCA PCS and PSR.

A reduction in arterial compliance has been linked with arterial stiffening from increased arterial collagen and calcification, reduced elastin content, and stiffening of vascular smooth muscle cells (Qiu et al. 2010; Fleenor et al. 2010). However, maintaining PA and exercise participation into later life may help to preserve the arterial wall from the common symptoms of arterial aging. For example, it has been argued that regular exercise can influence arterial remodeling as exercise induced increases in pulse pressure and arterial distension could realign collagen fibers within the arterial wall, which may alleviate arterial stiffening and improve arterial distension (Tanaka et al. 2000). Moreover, elastin content has also been shown to increase, and collagen content decrease in the large arteries of exercise trained aged rats (Gu et al. 2014). The greater levels of CCA PCS and PSR within the active group could help to buffer carotid artery blood flow, which possibly explains the lower resting CCA flow in the present study. In theory, this may defend the cerebral vasculature and tissue from damage caused by high pulsatile carotid blood flow (Mitchell et al. 2011), and potentially cushion the artery during periods of physiological stress, where increases in pulse pressure and cerebral blood flow pulsatility occur (Rosenberg et al. 2020). Whilst our data conform with previous reports that PCS and PSR was higher in individuals with high fitness levels or who were endurance trained (Pugh et al. 2018; Talbot et al. 2020), higher CCA PI was associated with greater PCS and PSR at rest, suggesting that PI in the CCA may not be alleviated with superior arterial compliance. However, it could be speculated that greater PCS and PSR in the active cohort may have alleviated ICA PI, which unlike CCA PI, was similar between groups at the P < 0.05 level. As this is the first study to assess CCA PCS and PSR alongside CCA PI and flow, further research in this area is therefore warranted to confirm such findings. Furthermore, as measures of blood velocity were not collected during or after the IHG protocol, it is not yet possible to determine whether differences in CA blood flow profiles or PI may have occurred between groups during physiological stress.

Despite observing a significant main between group difference in PCS and PSR, our data show a lack of time, or time*group interaction effect before, during and immediately after the 3-min IHG exercise. Although HR and BP within each group increased during and after the isometric HG exercise, it is possible that our cohort experienced lower exertions of physiological stress during the HG exercise compared to previous studies. For example, an isometric double leg press at 30% and 60% of maximal effort was implemented by Black et al. (2016), which not only consisted of a higher load (60% leg press vs 40% HG) but also involved larger muscle groups compared to the present study. Consequently, the participants performing the double leg press experienced a much greater change in mean arterial pressure (MAP) during the exercise compared to the participants in the current study. Larger increases in MAP may have facilitated the declines reported in PCS and PSR within the males during the double leg-press exercise (Black et al. 2016), as engagement of collagen fibers are known to increase during periods of high pressure (Holzapfel et al. 2000). Conversely, the results of the present study contrast with previous reports as neither group experienced changes in PCS nor PSR between time points. However, due to limited existing studies assessing PCS and PSR with exercise, the exact mechanisms underlying decreases in compliance in response to physiological stress are yet to be understood.

Conventional measures of arterial stiffness increase with advancing age, which is believed to be attributed in part to a shift in the arterial wall collagen and elastin ratio (Arnett et al. 1994; Thiebaud et al. 2016). However, physically active post-menopausal females have demonstrated similar augmentation index (AI) and pulse wave velocity (PWV) values to an active pre-menopausal cohort, despite exhibiting higher BP (Tanaka et al. 1998). Furthermore, Shibata et al. (2018) identified that central PWV was significantly slower, and β lower in committed or competitive healthy older adults compared to their sedentary or casual exercising age-matched counterparts. Our results contrast with previous reports as neither conventional stiffness measure differed between the active and inactive cohorts, despite significant differences in CCA PCS and PSR occurring. Interestingly, the correlation analysis identified that PCS and PSR from all timepoints had a stronger relationship with aerobic fitness levels than β and E_P._ Previous reports have also stated that CCA circumferential strain imaging has a stronger association and greater sensitivity than conventional stiffness measures in identifying differences in arterial elasticity with age (Oishi et al. 2008; Bjallmark et al. 2010). As long-term exercise has shown to reduce arterial collagen and improve elastin content within central arteries (Koutsis et al. 1995; Fleenor et al. 2010; Gu et al. 2014), the present results suggest that strain imaging via ultrasound speckle tracking has greater sensitivity than conventional measures, in identifying possible exercise derived differences in the intrinsic properties of CA.

### Brachial artery

Our finding that brachial artery FMD did not differ between active and inactive middle-aged females contrasts with previous studies reporting higher FMD% in healthy active cohorts. Systematic reviews and meta-analyses have shown that long-term endurance-based exercise training benefits BA FMD% when compared to sedentary aged-matched individuals (Montero et al. 2014; Campbell et al. 2019). Such improvements are a result of a healthier endothelial profile; including lower levels of oxidative stress and the preservation of nitric oxide (Taddei et al. 2000). While the active females in the present study displayed greater aerobic capacity and were more physically active than the inactive group, unlike the cohorts included within the meta-analyses, they were not profiled as long-term endurance trained or athletes. Rather, our study has included ‘real-world’ study groups which are more relatable to the general population, thus providing greater ecological validity. Furthermore, both groups demonstrate FMD values which are higher than values typically reported for the same age group of healthy females elsewhere (6.6 –7 vs. 4% Skaug et al. (2013)), suggesting that both groups exhibited an advantageous brachial endothelial profile. Nevertheless, whilst long-term endurance training enhances brachial artery FMD, differences may not occur when non-athletic middle-aged populations with higher levels of aerobic capacity are compared to their inactive counterparts.

### Cognitive function

Increased exercise levels and aerobic capacity have been associated with superior cognitive function in middle-aged and older adults within a number of studies (Clarkson-Smith and Hartley 1989; Aichberger et al. 2010), including cognitively intact participants (Barnes et al. 2003). However, within the present study the inactive middle-aged females performed better in one test of executive function assessed via animal fluency, whilst no other differences were observed between groups. Although the animal fluency score in the active cohort was lower than the typical ‘cut-off’ score and may indicate cognitive impairment (Canning et al. 2004), it is argued that impairment cannot be detected via a ‘one-test fits all’ approach (Cullen et al. 2007). Instead, our participants represent a high-functioning cognitive cohort as scores attained on the majority of cognitive tests were higher than typical ‘cut-off’ scores for dysfunction (Tombaugh 2004; Leung et al. 2011; Schroeder et al. 2012; Zenisek et al. 2016).

The inactive participants also exhibited superior resting CCA flow and lower pulsatility index in comparison to the active participants; however, such variables were not associated with better executive functioning. Previous studies report superior cerebral blood velocity, blood flow, and brain volumes in older adults who have greater levels of cardiorespiratory fitness (Ainslie et al. 2008; Zimmerman et al. 2014), and PA levels (Colcombe et al. 2006), respectively. However, the results of the present study, albeit with lower sample sizes than the aforementioned reports, differ from these previous findings as: 1) ICA flow was similar between groups; 2) there were minimal differences in cognitive function between groups; and 3) higher ${\dot{\mathrm{V}}\mathrm{O}}_{2\mathrm{peak}}$ was associated with worse animal fluency performance.

Our results may suggest that increased PCS and PSR of the central vasculature may not be as critical for cognitive function as initially hypothesized, whereas other factors such as superior brain blood flow could have a greater influence on the regulation of cognitive function. On the other hand, the possible detrimental effects of lower CCA PCS and PSR within the inactive cohort could take longer to influence cognitive function and may not become apparent until older age. Changes in cognitive function are cumulative with age (Park et al. 2002), yet previous research has demonstrated that higher PA levels or cardiorespiratory fitness at mid-life and older age was associated with less cognitive decline and cognitive disease when followed up years later (Laurin et al. 2001; Yaffe et al. 2001; Barnes et al. 2003; Larson et al. 2006). A longitudinal assessment is, therefore, needed to better understand whether lower CCA PCS and PSR seen within the inactive middle-aged females negatively impacts cognitive function into old age.

While other studies have mostly focused on the influence of exercise on either vascular or cognitive function, none have investigated the effects of exercise, CCA PCS, PSR, and cognitive function, together. It was initially hypothesized that increased PCS and PSR would benefit cognitive function by buffering high pressured blood flow and maintaining a constant steady flow toward the brain. However, the results suggest that enhanced distension (PCS) and speed of motion of the arterial wall (PSR) may not impact cognitive function in a group of physically active healthy middle-aged females. As there were a lack of differences within many of the physiological characteristics measured, the results may indicate that increased distension and arterial wall elasticity alone cannot affect cognition. Alternatively, improvements in cognition may only be seen when in combination with other physiological factors, such as increased ICA blood flow, improved FMD and potential reductions in oxidative stress. Furthermore, as our study included an exclusively healthy cohort, comparisons with unhealthy cohorts may also provide further insight into additional physiological mechanisms potentially linked with PCS and PSR. However, further research is required to better understand the influence of CCA PCS and PSR on cognitive function.

### Limitations

The present study contained some limitations. As the design of the study was cross-sectional, it cannot be guaranteed that the differences identified in CCA PCS and PSR are exclusively due to differences in PA levels and/or aerobic fitness between the groups. Furthermore, we did not collect information about the duration of physical activity engagement via the self-report questionnaire. Thus, we cannot determine the duration and intensity of activity required to achieve the superior arterial compliance observed within the active cohort of this study. Further work with objective assessment of physical activity may provide further insight to these factors and their associations with CCA mechanics.

Whilst participants verbally confirmed post-menopausal status, we did not collect menopause questionnaire data which could have provided information including duration since last menses. Blood analysis would have also been beneficial to confirm endogenous hormone concentration and confirm post-menopausal status.

We have also reported that different ultrasound machines were used to collect BA FMD data from the active and inactive participants. Although this was unavoidable due to equipment availability at the time of testing, attempts were made to standardize ultrasound image acquisition and data collection by following a number of guidelines from the most recent FMD expert consensus publication (Thijssen et al. 2019). Furthermore, all BA scans were analyzed using the same edge-detect software which is also recommended within the expert consensus publication to enhance study quality (Thijssen et al. 2019).

We also did not collect measurements of CA blood velocity during and immediately after the acute IHG exercise. Measurement of these data and the calculation of pulsatility index during and after the IHG exercise may have helped identify whether the differences in CCA PCS and PSR between groups affected blood flow directed towards the cerebrovascular system. Additionally, whilst initially hypothesized that the collected vascular measures would influence cognitive performance, all vascular measures were collected separately from the cognitive assessment. Measurement of vascular function and BP during the cognitive assessment may provide information about whether vascular dynamics influence cognitive performance when neurovascular coupling is elevated. However, the feasibility of these measurements would likely rely on the use of a non-verbal cognitive assessment, particularly when assessing the CA using ultrasound. Future studies should also consider including a young female comparison group to identify if active middle-aged healthy females preserve vascular and cognitive function, even in the absence of observed differences with their inactive or fully sedentary counterparts.

### Conclusion

This study is the first to report that active healthy middle-aged females possess greater CCA PCS, and PSR compared to their inactive counterparts. However, other than higher CCA flow and pulsatility index in the inactive group, this study failed to identify other differences in carotid or brachial artery structure and function, or most cognitive outcomes between the groups. Furthermore, there appears to be no linear relationship between CCA PCS and PSR with blood flow with cognitive function in healthy middle-aged females. Therefore, although regular participation in PA and exercise can reduce selective areas of vascular aging, such preservations may not necessarily benefit cognitive function, albeit when measured in a small sample of cognitively intact middle-aged females. Nevertheless, as middle-aged and post-menopausal females are at a high risk of vascular dysfunction, regular participation in PA and exercise is encouraged to help delay the progress of carotid arterial aging.

## Data Availability

Data supporting the study findings are available from the corresponding author on reasonable request.

## Abbreviations

ANOVA: Analysis of variance
β: Beta-stiffness index
BP: Blood pressure
CA: Carotid artery
CCA: Common carotid artery
DBP: Diastolic blood pressure
DiamDIAS: Diameter of artery during diastole
DiamSYS: Diameter of the artery during systole
ECG: Electrocardiogram
Ep: Peterson’s elastic modulus
FMD: Flow-mediated dilation
HR: Heart rate
HT: Hormone therapy
ICA: Internal carotid artery
IHG: Isometric handgrip
IMT: Intima-media thickness
NO: Nitric oxide
PA: Physical activity
PCS: Peak circumferential strain
PSR: Peak strain rate
PTT: Pulse transit time
SBP: Systolic blood pressure
${\dot{\mathrm{V}}\mathrm{O}}_{2\mathrm{peak}}$: Peak oxygen uptake
